# AI based prediction of post prostatectomy urinary incontinence and its impact on quality of life: development and validation study

**DOI:** 10.1038/s41598-026-51301-8

**Published:** 2026-05-25

**Authors:** Adamos Hadjivasiliou, Anand Kelkar, Ashwin Sridhar, Greg Shaw, Justin Collins, Prasanna Sooriakumaran, Senthil Nathan, Tim Briggs, John D. Kelly, Zafer Tandogdu, Ivana Drobnjak

**Affiliations:** 1 Hawkes Institute, University College London, London, UK; 2 University College London Hospitals NHS Foundation Trust, London, UK; 3 Division of Surgery and Targeted Intervention, University College London, London, UK; 4 Department of Computer Science, University College London, London, UK

**Keywords:** Diseases, Urology

## Abstract

Urinary incontinence can affect up to 53% of patients after radical prostatectomy, yet prediction tools rely on binary outcomes, missing heterogeneous recovery patterns. We developed machine-learning models that for the first time predict incontinence presence, severity, and quality-of-life impact at 3 and 12 months post-surgery. XGBoost models were trained on 21 perioperative features from 2,586 patients (2018–2021) and validated on 728 patients (2022). For patients incontinent at 3 months (n = 962), 12-month predictions achieved strong performance (AUC 0.82–0.86), with early quality-of-life impact (SHAP 1.4) outperforming symptom severity (SHAP 1.0) as a predictor. Three-month predictions using only baseline features showed moderate performance (AUC 0.52–0.59). Patients with identical total ICIQ-UI scores demonstrated divergent recovery trajectories when symptom severity and quality-of-life components were analyzed separately, with some achieving continence while others remained severely affected. Decomposing outcomes into multidimensional components rather than using aggregate scores enables personalized prediction of continence recovery, facilitating targeted counseling and rehabilitation strategies.

## Introduction

Post-prostatectomy incontinence (PPI) is a complex and multidimensional issue that significantly impacts patient quality of life (QoL)^1^. Conventional methods of assessment rely primarily on binary definitions, such as pad-free and/or leak-free status, which fail to capture the full spectrum of continence recovery^2^. While numerical pad usage is a simple and reproducible method to report PPI, it is influenced by individual habits and is subject to both patient and surgeon bias ^3^. The best way to report outcomes is by using validated tools designed for patients, i.e., patient-reported outcome measures (PROMs)^3^. Nevertheless, these tools aggregate multiple dimensions into a single score, which obscures critical nuances in patient experiences and introduces subjective variability^4^. These simplifications neglect crucial factors such as leakage frequency, volume, patient adaptation, and subjective experiences. Therefore, an approach that incorporates the multidimensional aspects of PPI should be adopted over binary classification. Advances in artificial intelligence (AI) provide an opportunity to capture this multidimensionality.

Improving our ability to predict PPI is crucial for optimizing presurgical counseling and postoperative management. Currently, patients considering robot-assisted radical prostatectomy (RARP) face confusing and inconsistent data, with reported 12-month PPI rates ranging from 13 to 53%^2^. While nomograms are used for counseling, they are developed with conventional statistical methods that rely on linear relationships, limiting their ability to model the complex interactions underlying incontinence^5,6^. In contrast, AI models excel at processing large, diverse datasets to identify intricate patterns, and several studies have demonstrated their potential for higher accuracy in predicting PPI compared to conventional methods^5,7^.

Recent advances in artificial intelligence have significantly enhanced predictive modeling in oncology, particularly in urological malignancies. A growing body of evidence supports the integration of machine learning algorithms to improve risk stratification and clinical decision-making. For instance, recent studies have demonstrated how AI-based approaches can outperform traditional statistical models in predicting oncological outcomes and identifying high-risk patients, highlighting their potential role in precision medicine. These findings further support the clinical relevance of implementing AI-driven predictive tools in routine practice^8^.

Despite this potential, the application of AI in predicting PPI is hindered by several limitations. Most studies define incontinence using a binary classification based on pad usage, which fails to capture the full spectrum of its severity or its broader impact on patient QoL^9^. Furthermore, many models are developed using small sample sizes (100–848 patients) and focus primarily on short-term outcomes, leaving a gap in our understanding of long-term recovery^5,7^. Structurally, AI models often face challenges such as imbalanced datasets and single-institution training, which reduce their reliability across diverse patient populations^5,7,10^. To improve predictive precision and clinical utility, future AI models must incorporate multidimensional patient experiences, standardized data collection, and robust validation.

In this study, we introduce a novel AI framework designed to address the unmet need for precise and multidimensional prediction of PPI. Unlike conventional models that rely on binary outcomes or aggregate scores, our approach independently predicts incontinence status, severity of physical symptoms, and impact on QoL, thereby capturing the full spectrum of patient experiences. Using a large, high-quality dataset from over 2000 RARP cases at University College London Hospitals, and leveraging validated PROMs, we developed machine learning models that generate short and long-term predictions. This multidimensional predictive system has the potential to inform individualized recovery strategies and support shared decision-making in prostate cancer care.

## Methods

Our study utilized a dataset of 2586 patients who underwent RARP at UCLH between 2018 and 2021. We extracted 21 features, including pre-surgical clinicopathological data, surgery-related information, and post-surgical self-reported incontinence data collected via the International Consultation on Incontinence Questionnaire—Urinary Incontinence Short Form (ICIQ-UI-SF) at 3 and 12 months. Using XGBoost classifiers, we developed models to predict four outcome features: Incontinence Status, Overall incontinence Severity, Symptoms Severity, and Impact on QoL. We created short-term (3-month) and long-term (12-month) prediction models, with the latter incorporating additional 3-month post-surgery data. Model performance was evaluated using various metrics, including accuracy, precision, recall, F1-score, and AUC-ROC, while SHAP values were employed to enhance model interpretability. The respective 2022 data was used for validation (777 patients).

### Data collection and pre-processing

This study was conducted in accordance with the Declaration of Helsinki and approved by the University College London Hospitals NHS Foundation Trust Research Ethics Committee (R&D Reference: 173477). Individual patient consent was waived for this retrospective analysis of anonymized quality assurance data, in accordance with NHS Health Research Authority guidance. Clinical trial number: not applicable.

Data were collected prospectively by independent data coordinators as part of the ongoing quality assurance (QA) program for RARP services at University College London Hospitals NHS Foundation Trust. Summary statistics from these data are reviewed internally on a quarterly basis. Data validation was led by the assigned data coordinator in collaboration with the QA lead (ZT), following standardized protocols previously published^11^.

All patients who underwent primary RARP at UCLH between January 2018 and December 2021 were eligible for inclusion. Salvage prostatectomy cases (n = 106) and patients without complete continence follow-up data at 3 months (n = 268) were excluded. After exclusions, 2,212 patients with complete 3-month follow-up were included in the analysis (see Appendix, Fig. 11 for patient case disposition).

**Fig. 1:**
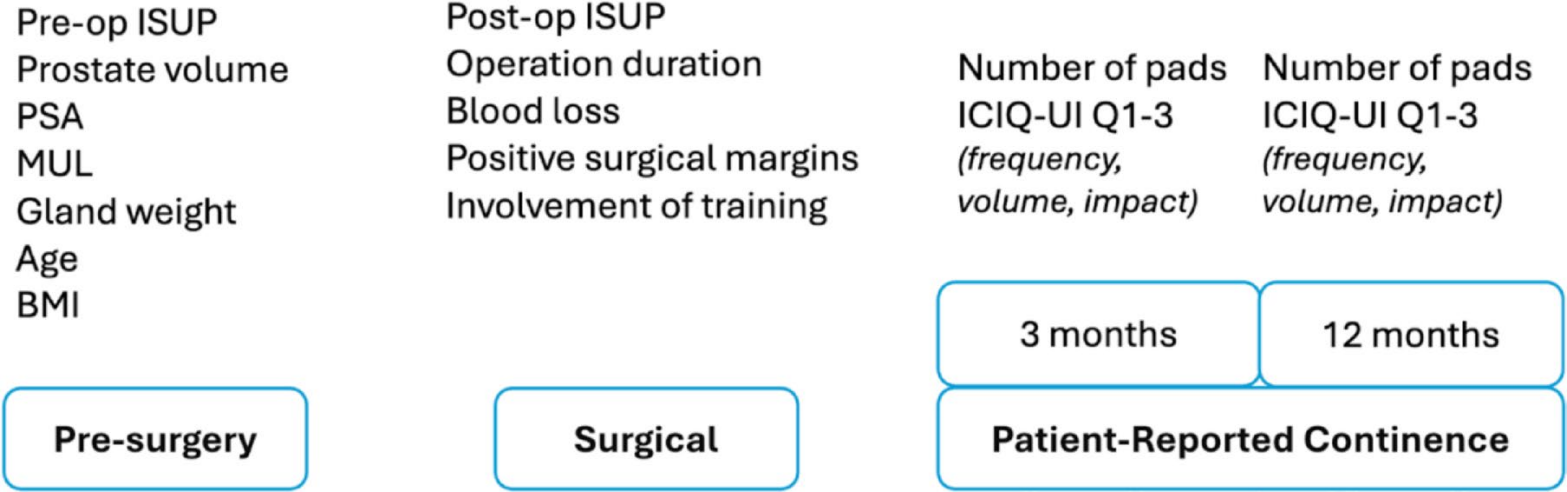
List of available features obtained from the UCLH quality assurance audit data. The ICIQ-UI score (range 0–21) combines frequency (0–5, Q3), volume (0–6, Q4), and impact (0–10, Q5) of urinary incontinence. Surgeon grade indicates if the operation was carried out by a consultant surgeon or a fellow under the direct observation of a consultant. Nerve-sparing status was categorized by lobe involvement as bilateral, uni lateral, or none. (abbreviations used; BMI = Body mass index, ISUP: International Society of Urological Pathology, MUL = Membranous urethral length, ICIQ-UI = International Consultation on Incontinence Questionnaire—Urinary Incontinence).

In total 21 features were extracted from the dataset and categorized into: clinico pathological features before surgery, surgery-related features, and self-reported incontinence data at 3 and 12 months post-surgery (Fig. 1).

Pre-surgery features included pre-op ISUP (International Society of Urological Pathology) grade, prostate volume, PSA (Prostate-Specific Antigen), MUL (Membranous urethral length), age, and body mass index (BMI). MUL is obtained through MRI of the prostate defined as the distance between the apex of the prostate and the level of the urethra enters the penile bulb. At UCLH all measurements were made by expert uro-radiologists.

Surgical features comprised post-operative ISUP grade, nerve-sparing status (none, unilateral, bilateral, unknown), operation duration, blood loss, and involvement of training (No = Consultant, Yes = fellow). Missing data in baseline or surgical features were imputed using k-Nearest Neighbors (k = 5) with Euclidean distance. A table detailing the percentage of missing data for each feature can be found in the Appendix Table 5. MUL had the highest missingness rate among all baseline features at 64%. A sensitivity analysis confirmed that excluding MUL entirely did not substantially alter model performance (see Results).

**Table 1:** Patient demographics and clinical characteristics by Incontinence Status, Symptoms Severity, and QoL impact at three months post-operation. Continuous features are presented as mean values ± standard deviation, categorical features as count (percentage %). Corresponding p-values for statistical significance were calculated using Pearson correlation coefficient for continuous variables and chi-squared test of independence for categorical variables.

Feature	Continence status			Symptoms severity			Impact on QoL		
	Mean values		p-value	Mean values		p-value	Mean values		p-value
	Pad free (n = 1250)	At least 1 pad (n = 962)		Low (n = 1250)	High (n = 962)		Low (n = 1765)	High (n = 447)	
BMI (kg/m^2^)	27.81 ± 4.13	27.50 ± 3.88	0.26	27.81 ± 4.13	27.50 ± 3.88	0.26	27.52 ± 3.86	28.06 ± 4.44	0.09
MUL (mm)	15.36 ± 3.26	13.78 ± 3.12	< 0.01	15.36 ± 3.26	13.78 ± 3.12	< 0.01	15.07 ± 3.26	13.30 ± 3.04	< 0.01
PreOp PSA (ng/mL)	8.92 ± 6.82	9.76 ± 14.44	0.20	8.92 ± 6.82	9.76 ± 14.44	0.20	9.21 ± 11.46	9.58 ± 8.03	0.34
MRI prostate volume (cc)	40.46 ± 18.88	46.73 ± 29.52	0.07	40.46 ± 18.88	46.73 ± 29.52	0.07	41.56 ± 20.20	48.39 ± 34.13	0.18
Age (years)	62.14 ± 6.93	63.92 ± 7.22	< 0.01	62.14 ± 6.93	63.92 ± 7.22	< 0.01	62.57 ± 6.90	64.27 ± 7.73	< 0.01
Measured blood loss (L)	0.15 ± 0.37	0.16 ± 0.38	0.51	0.15 ± 0.37	0.16 ± 0.38	0.51	0.15 ± 0.38	0.16 ± 0.38	0.65
Duration of operation (h)	2.82 ± 1.43	3.00 ± 1.38	0.01	2.82 ± 1.43	3.00 ± 1.38	0.01	2.85 ± 1.41	3.08 ± 1.37	0.01
ISUP PreOp grade									
Grade 1	142 (11.4%)	97 (10.1%)	0.37	142 (11.4%)	97 (10.1%)	0.37	188 (10.7%)	51 (11.4%)	0.71
Grade 2	736 (58.9%)	553 (57.5%)	0.54	736 (58.9%)	553 (57.5%)	0.54	1047 (59.3%)	242 (54.1%)	0.05
Grade 3	252 (20.2%)	201 (20.9%)	0.71	252 (20.2%)	201 (20.9%)	0.71	353 (20.0%)	100 (22.4%)	0.30
Grade 4	78 (6.2%)	66 (6.9%)	0.62	78 (6.2%)	66 (6.9%)	0.62	112 (6.3%)	32 (7.2%)	0.61
Grade 5	41 (3.3%)	45 (4.7%)	0.12	41 (3.3%)	45 (4.7%)	0.12	64 (3.6%)	22 (4.9%)	0.26
ISUP PostOp grade									
Grade 1	105 (8.4%)	56 (5.8%)	0.03	105 (8.4%)	56 (5.8%)	0.03	132 (7.5%)	29 (6.5%)	0.54
Grade 2	795 (63.6%)	607 (63.1%)	0.84	795 (63.6%)	607 (63.1%)	0.84	1141 (64.6%)	261 (58.4%)	0.02
Grade 3	270 (21.6%)	238 (24.7%)	0.09	270 (21.6%)	238 (24.7%)	0.09	382 (21.6%)	126 (28.2%)	< 0.01
Grade 4	19 (1.5%)	16 (1.7%)	0.92	19 (1.5%)	16 (1.7%)	0.92	26 (1.5%)	9 (2.0%)	0.54
Grade 5	59 (4.7%)	45 (4.7%)	1.00	59 (4.7%)	45 (4.7%)	1.00	82 (4.6%)	22 (4.9%)	0.90
Nerve sparing									
Bilateral	563 (45.0%)	359 (37.3%)	< 0.01	563 (45.0%)	359 (37.3%)	< 0.01	777 (44.0%)	145 (32.4%)	< 0.01
Unilateral	444 (35.5%)	316 (32.8%)	0.21	444 (35.5%)	316 (32.8%)	0.21	619 (35.1%)	141 (31.5%)	0.18
No nerve sparing	234 (18.7%)	275 (28.6%)	< 0.01	234 (18.7%)	275 (28.6%)	< 0.01	353 (20.0%)	156 (34.9%)	< 0.01
Unknown	9 (0.7%)	12 (1.2%)	0.30	9 (0.7%)	12 (1.2%)	0.30	16 (0.9%)	5 (1.1%)	0.89
Surgeon grade									
Consultant	907 (72.6%)	730 (75.9%)	0.09	907 (72.6%)	730 (75.9%)	0.09	1299 (73.6%)	338 (75.6%)	0.42
SpR	262 (21.0%)	183 (19.0%)	0.28	262 (21.0%)	183 (19.0%)	0.28	364 (20.6%)	81 (18.1%)	0.27

Patient-reported incontinence data at 3 and 12 months post-surgery were collected using the ICIQ-UI^12^, a standardized, validated questionnaire designed to measure the impact of urinary incontinence on a person’s life. Specifically, ICIQ-UI assesses frequency of urinary incontinence episodes, severity of urine leakage (how much urine is lost), impact of incontinence on daily life (including physical, social, and psychological effects) and type of incontinence symptoms (stress, urgency, or mixed incontinence)^13^.

The ICIQ-UI consists of three scored questions:

Q3) “How often do you leak urine?” Scored 0–5 points.

Q4) “How much urine do you usually leak (whether you wear protection or not)?” Scored 0, 2, 4, or 6 points.

Q5) “Overall, how much does leaking urine interfere with your everyday life?” Scored 0–10 points.

In addition to completing the ICIQ-UI questionnaire, patients reported on their average daily use of incontinence pads over the week prior to both the 3 and 12 month follow-ups. Patients who were continent at 3 months were not assessed again at 12 months.

### Defining the outcome variables

We define and analyze the following outcome measures:

- Incontinence: Defined by patient reported number of pads used per day over the preceding week. Patients are categorized as pad-free (pads = 0) or not pad-free (pads > 0).
- Overall ICIQ Severity: Assessed using the total ICIQ-UI score, calculated as the sum of questions 3, 4, and 5 (Q3 + Q4 + Q5), ranging from 0 to 21. Severity was classified into^14^: Continent (0), Mild/Moderate (1–12), and Severe (13–21).
- Symptoms Severity: Measured by Q3 + Q4, the sum of ICIQ-UI questions 3 (frequency of urine leak) and 4 (volume of urine leak) to obtain a composite score in the range 0–11. We define slight/Moderate Symptoms for score range (0–5) as low and Severe/very severe Symptoms (6–11) as high.
- Impact on daily life: Measured by Q5 of ICIQ-UI, which captures interference with daily life. This was again classified as low (1–5) and high (6–10).

**Fig. 2:**
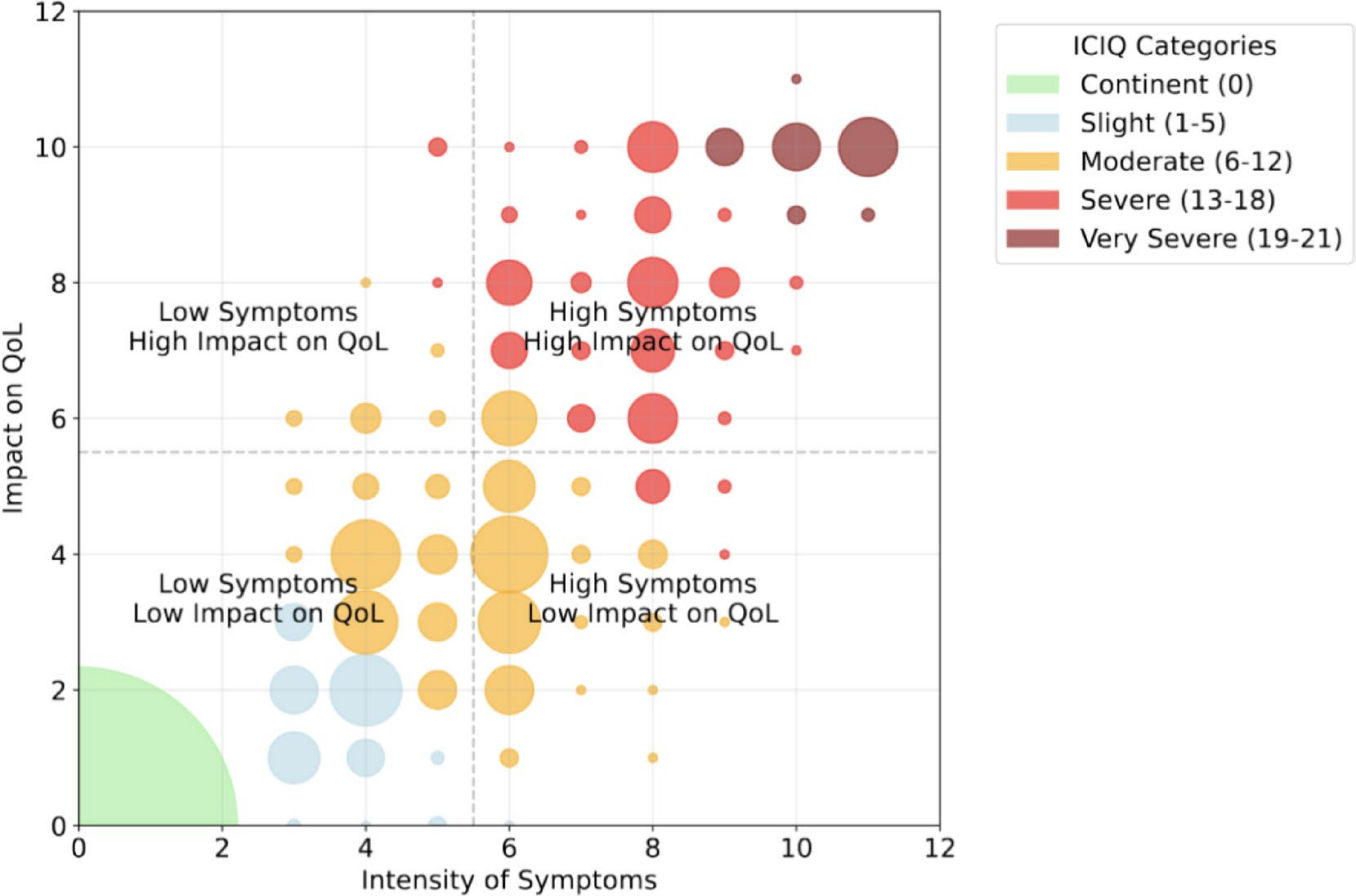
Multidimensional analysis of post-RARP urinary incontinence at 3 months. This bubble plot illustrates the distribution of patients across Symptom Severity and Impact on QoL, with bubble size representing patient count. Colors indicate ICIQ UI severity categories from Green (continent) to Red (very severely incontinent). The quadrant divisions highlight the nuanced patient experiences that may be overlooked by total ICIQ-UI scores alone. For analysis, Mild/Moderate and Sever/Very Severe categories were combined.

Figure 2 shows significant variation in patient experiences hidden by total ICIQ-UI scores alone. Patients with the same ICIQ severity category (shown by color) distribute differently across symptom severity and quality of life dimensions. For example, moderate severity patients (orange, ICIQ 6–12) appear in all four quadrants—some with high symptoms but low QoL impact, others showing the opposite. The lower left quadrant notably contains patients with minimal symptoms yet considerable QoL impact, suggesting factors beyond physical symptoms affect patient experience. These distinct patterns justify our multidimensional approach, as total ICIQ scores miss clinically important differences that may predict different recovery paths and need tailored interventions.

### Statistical analysis

Univariate associations between the collected data variables and the outcome variables (Continence Status, Overall Incontinence Severity, Symptoms Severity, Impact on QoL) at 3 and 12 months were assessed using Pearson correlation for continuous features and chi-square tests for categorical features. For continuous variables, correlation coefficients (r) were calculated to quantify the strength and direction of relationships, Statistical significance was set at p * < *0.05. All analyses were performed using Python 3.11, and Scikit-learn 1.5.0. Detailed patient demographics can be found in Tables 1 and 2.

**Table 2:** Patient demographics and clinical characteristics by incontinence status, Symptoms Severity, and QoL impact at twelve months post-operation. Continuous features are presented as mean values (± SD), categorical features as count (percentage %), and 3-month outcomes as median (± SD), with corresponding p-values for statistical significance.

Feature	Continence: Pad free (n = 685)	Continence: ≥ 1 pad (n = 277)	Continence p-value	Symptoms: Low (n = 695)	Symptoms: High (n = 267)	Symptoms p-value	QoL impact: Low (n = 800)	QoL impact: High (n = 162)	QoL impact p-value
Continuous features (mean ± SD)									
BMI (kg/m^2^)	27.76 ± 4.04	27.93 ± 4.35	0.58	27.77 ± 4.04	27.90 ± 4.38	0.69	27.70 ± 4.04	28.39 ± 4.53	0.12
MUL (mm)	13.84 ± 2.85	13.64 ± 3.65	0.57	13.88 ± 2.85	13.53 ± 3.70	0.32	13.81 ± 2.96	13.66 ± 3.65	0.80
PreOp PSA (ng/mL)	9.40 ± 7.05	8.96 ± 5.15	0.97	9.30 ± 6.91	9.11 ± 5.21	0.79	9.43 ± 6.88	8.72 ± 4.88	0.88
MRI prostate volume (cc)	45.91 ± 24.62	48.28 ± 37.26	0.80	45.46 ± 25.17	49.74 ± 37.97	0.54	46.57 ± 25.38	47.21 ± 39.78	0.39
Age (years)	63.36 ± 7.09	63.77 ± 8.22	0.18	63.36 ± 7.09	63.77 ± 8.22	0.18	63.47 ± 7.07	63.49 ± 9.18	0.45
Measured blood loss (L)	0.15 ± 0.38	0.18 ± 0.39	0.26	0.15 ± 0.38	0.18 ± 0.39	0.26	0.15 ± 0.38	0.21 ± 0.41	0.13
Duration of operation (h)	2.91 ± 1.33	3.21 ± 1.49	< 0.01	2.91 ± 1.33	3.21 ± 1.49	< 0.01	2.97 ± 1.36	3.17 ± 1.49	0.16
ISUP PreOp grade (n, %)									
Grade 1	65 (9.5%)	32 (11.6%)	0.40	68 (9.8%)	29 (10.9%)	0.71	72 (9.0%)	25 (15.4%)	0.02
Grade 2	408 (59.6%)	145 (52.3%)	0.05	413 (59.4%)	140 (52.4%)	0.06	474 (59.2%)	79 (48.8%)	0.02
Grade 3	134 (19.6%)	67 (24.2%)	0.13	135 (19.4%)	66 (24.7%)	0.09	165 (20.6%)	36 (22.2%)	0.73
Grade 4	49 (7.2%)	17 (6.1%)	0.67	49 (7.1%)	17 (6.4%)	0.82	55 (6.9%)	11 (6.8%)	1.00
Grade 5	29 (4.2%)	16 (5.8%)	0.39	30 (4.3%)	15 (5.6%)	0.49	34 (4.2%)	11 (6.8%)	0.23
ISUP PostOp grade (n, %)									
Grade 1	41 (6.0%)	15 (5.4%)	0.85	42 (6.0%)	14 (5.2%)	0.75	45 (5.6%)	11 (6.8%)	0.69
Grade 2	443 (64.7%)	164 (59.2%)	0.13	451 (64.9%)	156 (58.4%)	0.07	509 (63.6%)	98 (60.5%)	0.51
Grade 3	160 (23.4%)	78 (28.2%)	0.14	161 (23.2%)	77 (28.8%)	0.08	195 (24.4%)	43 (26.5%)	0.63
Grade 4	10 (1.5%)	6 (2.2%)	0.62	10 (1.4%)	6 (2.2%)	0.55	13 (1.6%)	3 (1.9%)	1.00
Grade 5	31 (4.5%)	14 (5.1%)	0.85	31 (4.5%)	14 (5.2%)	0.73	38 (4.8%)	7 (4.3%)	0.97
Nerve sparing (n, %)									
Bilateral	261 (38.1%)	98 (35.4%)	0.47	264 (38.0%)	95 (35.6%)	0.54	300 (37.5%)	59 (36.4%)	0.86
Unilateral	228 (33.3%)	88 (31.8%)	0.71	232 (33.4%)	84 (31.5%)	0.62	268 (33.5%)	48 (29.6%)	0.39
Unknown	12 (1.8%)	0 (0.0%)	0.06	12 (1.7%)	0 (0.0%)	0.07	12 (1.5%)	0 (0.0%)	0.24
Surgeon grade (n, %)									
Consultant	526 (76.8%)	204 (73.6%)	0.34	532 (76.5%)	198 (74.2%)	0.49	610 (76.2%)	120 (74.1%)	0.62
SpR	122 (17.8%)	61 (22.0%)	0.16	125 (18.0%)	58 (21.7%)	0.22	150 (18.8%)	33 (20.4%)	0.71
3-month outcomes (median ± SD)									
Symptoms severity (3 months)	6.00 ± 1.87	8.00 ± 2.15	< 0.01	6.00 ± 1.86	8.00 ± 2.15	< 0.01	6.00 ± 1.98	8.00 ± 1.93	< 0.01
Impact on QoL (3 months)	4.00 ± 2.44	8.00 ± 2.72	< 0.01	4.00 ± 2.45	8.00 ± 2.73	< 0.01	4.00 ± 2.56	8.00 ± 2.41	< 0.01
ICIQ score (3 months)	1.00 ± 0.79	2.00 ± 1.42	< 0.01	1.00 ± 0.79	3.00 ± 1.43	< 0.01	1.00 ± 0.87	3.00 ± 1.52	< 0.01

### Model development

**Fig. 3:**
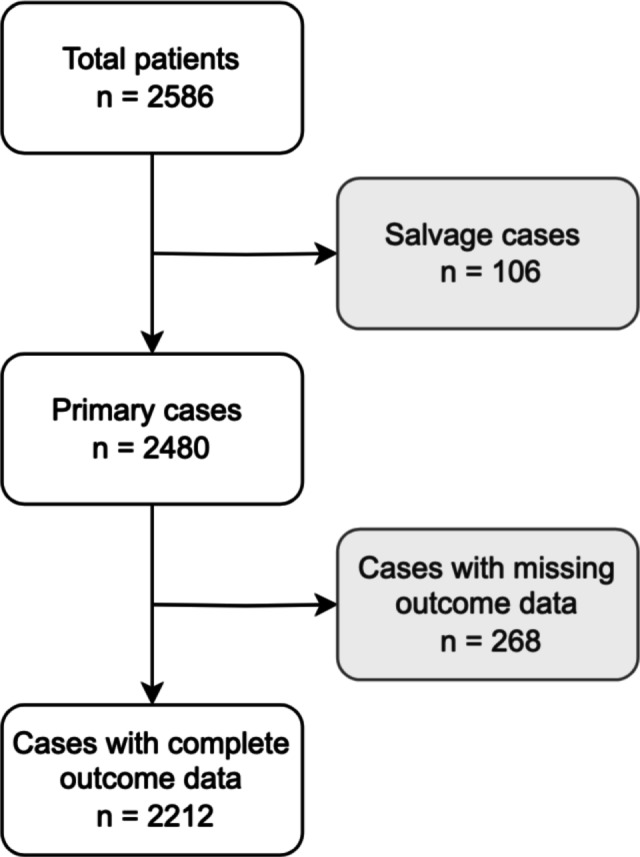
Patient selection and model development flowchart. From the UCLH database (2586 patients), after preprocessing two cohorts were created: a 3-month group and a 12-month group (962 patients, 29% with urinary incontinence based on pad usage). Both cohorts were split 70/30 for training and validation. For analysis, Mild/Moderate and Sever/Very Severe categories were combined.

The patient selection process and model development workflow are illustrated in Fig. 3, with filtering in Appendix Fig. 11. We developed two sequential prediction models to capture different stages of recovery, with each model predicting four outcome variables: Incontinence Status, Overall ICIQ Severity, Symptom Severity, and Impact on QoL. Our modeling approach consisted of two distinct prediction scenarios:

*Short-term prediction (3 months):* Using only preoperative clinicopathological data and intraoperative surgical variables, we predicted outcomes at 3 months post-surgery. This model was trained on all 2,212 patients, of whom 41.3% (n = 914) experienced incontinence at 3 months.

*Long-term prediction (12 months):* For patients who were incontinent at 3 months, we predicted their 12-month outcomes by incorporating their 3-month postoperative data (ICIQ scores, pad usage) alongside their baseline and surgical variables. This model focused exclusively on the 962 patients with incontinence at 3 months, among whom 29% (n = 279) remained incontinent at 12 months, while 71% achieved continence.

We evaluated multiple algorithms including Random Forest, Support Vector Machines, and neural networks during model development, but XGBoost consistently demonstrated superior performance across all outcome measures. Given our focus on clinical application and interpretability rather than algorithmic comparison, we proceeded with the best-performing model to maximize predictive accuracy and enable meaningful SHAP-based feature importance analysis.

Categorical features (nerve-sparing status, surgeon grade, ISUP grade) were one-hot encoded. Continuous features were standardised using z-score scaling. Missing values were imputed using k-Nearest Neighbours (k = 5, Euclidean distance). For binary outcomes, class imbalance was addressed by tuning XGBoost’s scale_pos_weight parameter proportionally to the inverse class ratio. The dataset was split into a stratified 70/30 training/testing set. Hyperparameter tuning was performed via grid search with fivefold stratified cross-validation on the training set. All features were included a priori based on clinical relevance; no automated feature selection was applied.

External validation was performed using data from 728 patients treated in 2022 at UCLH, with models tested both in their original form and after fine-tuning on 25% of the validation cohort (stratified by outcome) to assess adaptability to temporal changes in surgical practice and patient populations.

### Model evaluation and interpretability

Performance for each model was evaluated using accuracy, precision, recall, F1-score, and AUC-ROC. These metrics provide complementary insights: accuracy for overall correctness, precision and recall for false positive and negative rates, while AUC ROC assesses discriminative ability across different classification thresholds. All performance metrics are reported with 95% confidence intervals computed via bootstrap resampling (1000 iterations, percentile method). Model calibration was assessed using calibration plots and Brier scores for all binary models.

To enhance interpretability, SHAP values were employed. SHAP leverages game theory to attribute the contribution of each feature to individual predictions, offering a detailed breakdown of feature importance and interactions. This approach allows for both global interpretation of the model’s behavior across the entire dataset and local interpretation for individual predictions.

## Results

We analyzed outcomes from 2,212 patients who underwent RARP between 2018 and 2021. We found that early prediction remains challenging despite advanced machine learning, but incorporating 3-month data significantly improves 12-month predictions. Notably, patients with identical ICIQ scores showed markedly different recovery trajectories when symptom severity and quality of life impact were analyzed separately.

### Short-term prediction (3 months)

We achieved moderate predictive performance for 3-month outcomes, with age, BMI, and MUL emerging as the strongest predictors. Our models reached AUC-ROC values of 0.52–0.59 across different outcome measures.

#### Patient demographics

2212 patients were reviewed at 3 months of which 41% were incontinent (= > 1 pad). Symptom severity and impact on QoL ranged from 0 to 11. The demographics are presented in Table 1. BMI showed a significant gradient across QoL impact groups (p = 0.03), with higher BMI (28.05 ± 4.44) associated with greater impact on QoL compared to the low-impact group (27.52 ± 3.86). MUL demonstrated consistent and highly significant associations (p < 0.01) across all outcome measures. Age demonstrated a strong and consistent relationship with outcomes (p < 0.01 across all measures), with older patients more frequently represented in adverse outcome categories. The duration of operation also emerged as a significant factor (p < 0.01), with longer operative times associated with poorer outcomes across all measures.

The nerve-sparing approach showed particularly strong associations with outcomes (p < 0.01), with bilateral nerve-sparing procedures representing 45.04% of pad-free cases compared to 37.32% of cases requiring pads. This disparity was even more pronounced in QoL impact, where bilateral nerve-sparing represented 44.02% of low impact cases but only 32.44% of high-impact cases. Involvement of training did not show significant associations with outcomes (p > 0.05).

Descriptive statistics for ICIQ Severity can be found in Appendix Table 6.

#### Model results and evaluation

**Fig. 4:**
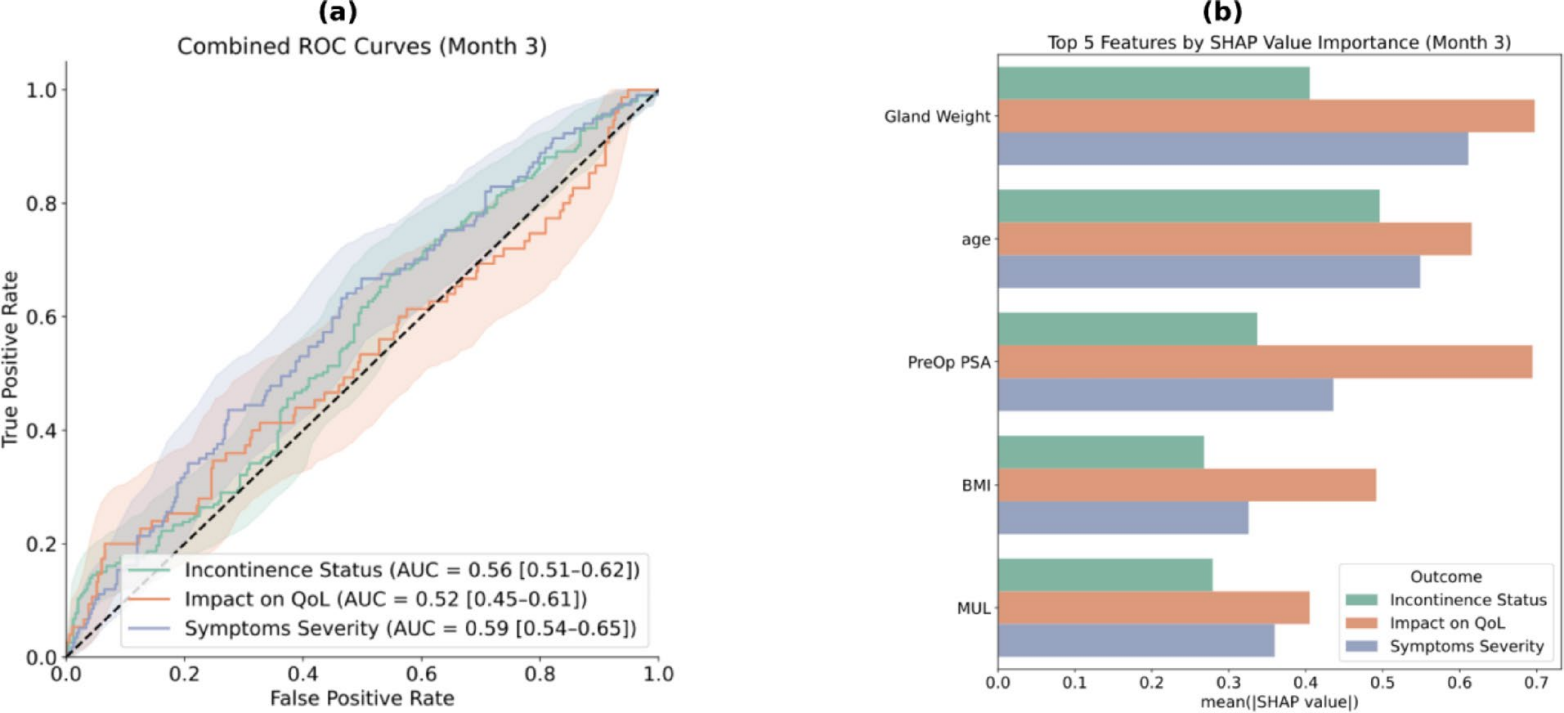
The 3-month prediction model performance through ROC curves for Incontinence Status (AUC = 0.56 [0.51–0.62]), Impact on QoL (AUC = 0.52 [0.45–0.61]), and Symptoms Severity (AUC = 0.59 [0.54–0.65]), along with the corresponding SHAP values showing feature importance.

**Fig. 5:**
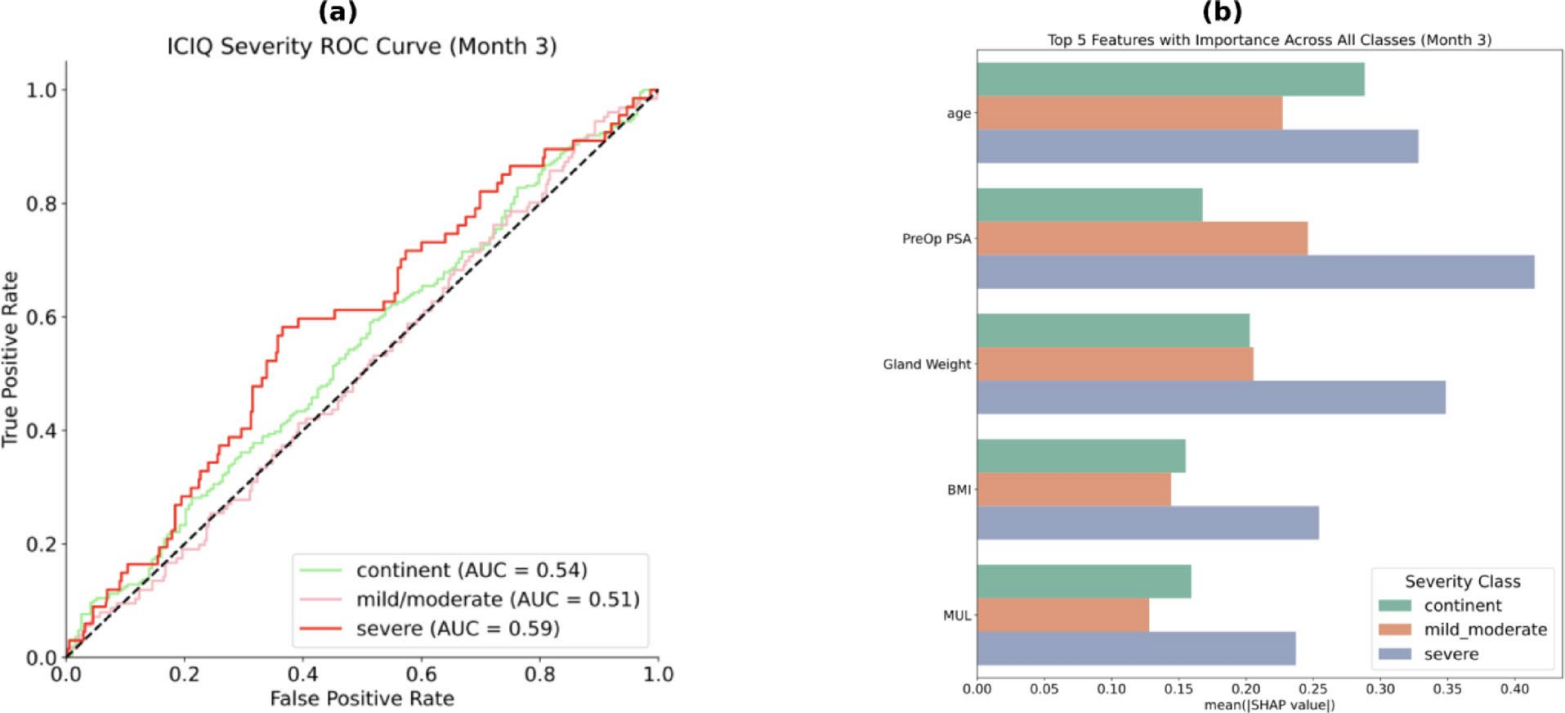
The ROC curves for different ICIQ severity categories at 3 months: continent (AUC = 0.54), mild/moderate (AUC = 0.51), and severe (AUC = 0.59) ICIQ severity classes at 3 months, along with the SHAP values showing feature importance across all severity classes.

The short-term prediction model for ICIQ-UI score severity at 3 months post-surgery demonstrated varying performance across different classification tasks (Figs. 4 and 5). For the binary classification of Incontinence Status, the model achieved an AUC-ROC of 0.56 (95% CI: 0.51–0.62). The Symptoms Severity and Impact on QoL models showed AUC-ROC values of 0.59 (95% CI: 0.54–0.65) and 0.52 (95% CI: 0.45–0.61), respectively (Fig. 4a).

The Symptoms model achieved accuracy of 0.664, precision of 0.361, recall of 0.342, and F1-score of 0.350, while the Impact model showed accuracy of 0.774, precision of 0.271, recall of 0.200, and F1-score of 0.229. The mean absolute SHAP values for the top five features are: Gland Weight, age, PreOp PSA, BMI, and MUL (Fig. 4b). Age and BMI had a higher association on Impact on QoL than Symptoms severity.

Calibration analysis can be seen in Appendix Figs. 12–17.

### Long-term prediction (12 months)

We focused on the 962 patients who were incontinent at 3 months, finding that 71% achieved continence by 12 months. Adding 3-month outcome data to our models improved prediction accuracy substantially, with AUC-ROC values reaching 0.82–0.86. Early QoL impact proved more predictive of long-term recovery than early symptom severity itself.

#### Patient demographics

At 12 months, we assessed urinary continence in the 962 patients who were incontinent (≥ 1 pad) at 3 months. As shown in Table 2, 685 patients (71.2%) were pad-free, while 277 (28.8%) continued to use at least one pad per day. At 12 months, patients who were pad-free (n = 685) had significantly shorter operative times compared to those still using pads (2.91 vs. 3.21 h, p < 0.01). BMI, MUL, preoperative PSA, prostate volume, age, blood loss, nerve-sparing status and surgeon grade (Consultant vs. Fellow) were not significantly associated with continence or symptom outcomes. Patients with high symptom severity or high QoL impact at 12 months had consistently worse 3-month outcomes, including higher symptom severity scores, greater interference with daily life, and higher ICIQ-UI scores (all p < 0.01). These findings suggest that early post-operative symptom burden may be predictive of longer-term incontinence and its impact on quality of life. Results for ICIQ Severity can be found in Appendix Table 7.

**Table 3:** Model performance across original training (2018–2021), external validation (2022), and fine-tuned validation (2022), showing F1-scores with AUC-ROC in parentheses for various outcomes at 3- and 12-month prediction intervals.

Model	Time	Original training	External validation	Fine-tuned validation
Binary	3 m	**0.48**(0.56)	0.39 (0.52)	0.31 (0.45)
Symptoms	3 m	**0.35**(0.59)	0.18 (0.55)	0.22 (0.45)
Impact	3 m	0.23 (0.53)	0.26 (0.53)	0.27 (0.53)
Severity	3 m	**0.45**(0.54)	0.51 (0.54)	0.52 (0.50)
Binary	12 m	**0.63**(0.83)	0.65 (0.61)	**0.72**(0.77)
Symptoms	12 m	**0.51**(0.82)	0.32 (0.78)	0.46 (0.88)
Impact	12 m	**0.53**(0.86)	0.21 (0.58)	0.40 (0.85)
Severity	12 m	**0.68**(0.76)	0.32 (0.52)	**0.66**(0.57)

#### Model results and evaluation

**Fig. 6:**
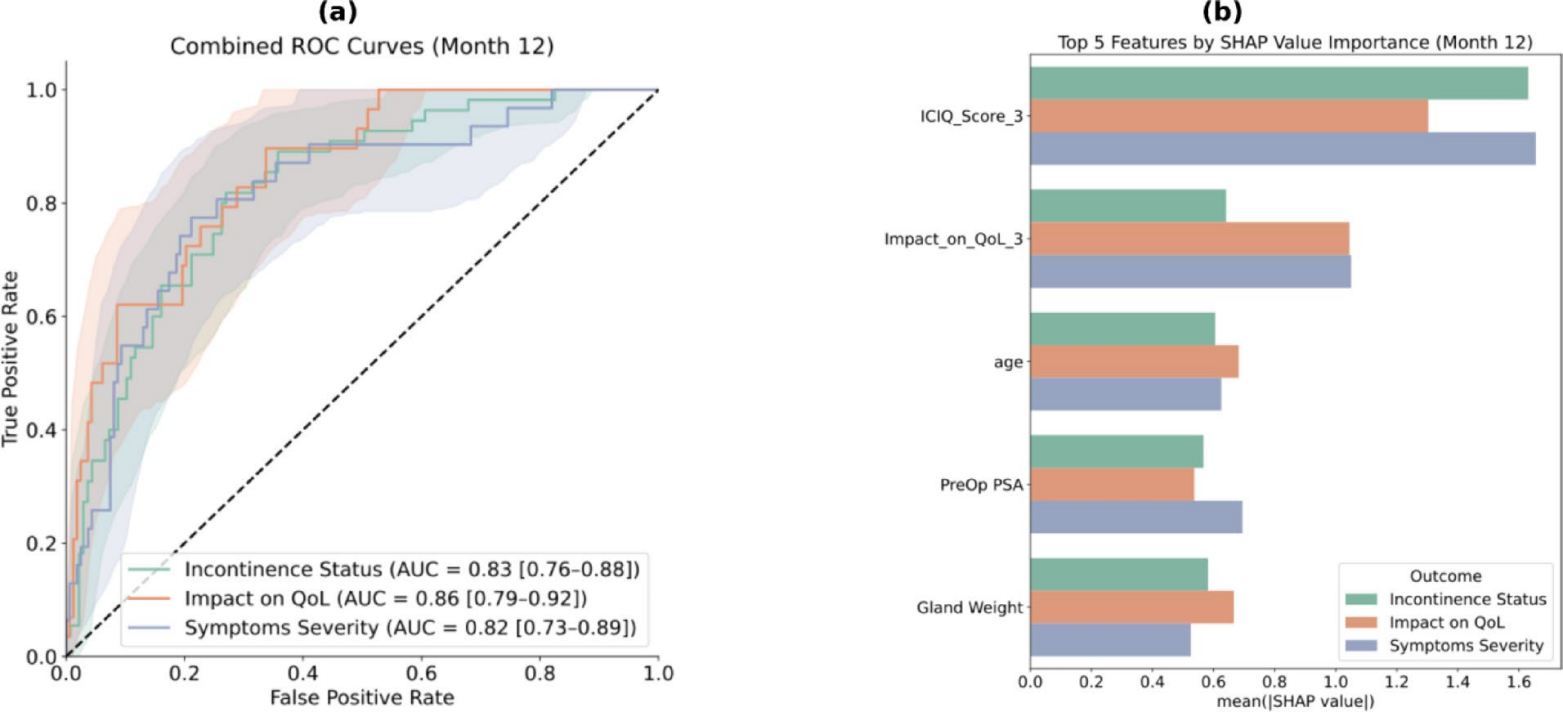
The 12-month prediction model performance through ROC curves for Incontinence Status (AUC = 0.83 [0.76–0.88]), Impact on QoL (AUC = 0.86 [0.79–0.92]), and Symptoms Severity (AUC = 0.82 [0.73–0.89]), along with the corresponding SHAP values showing feature importance.

**Fig. 7:**
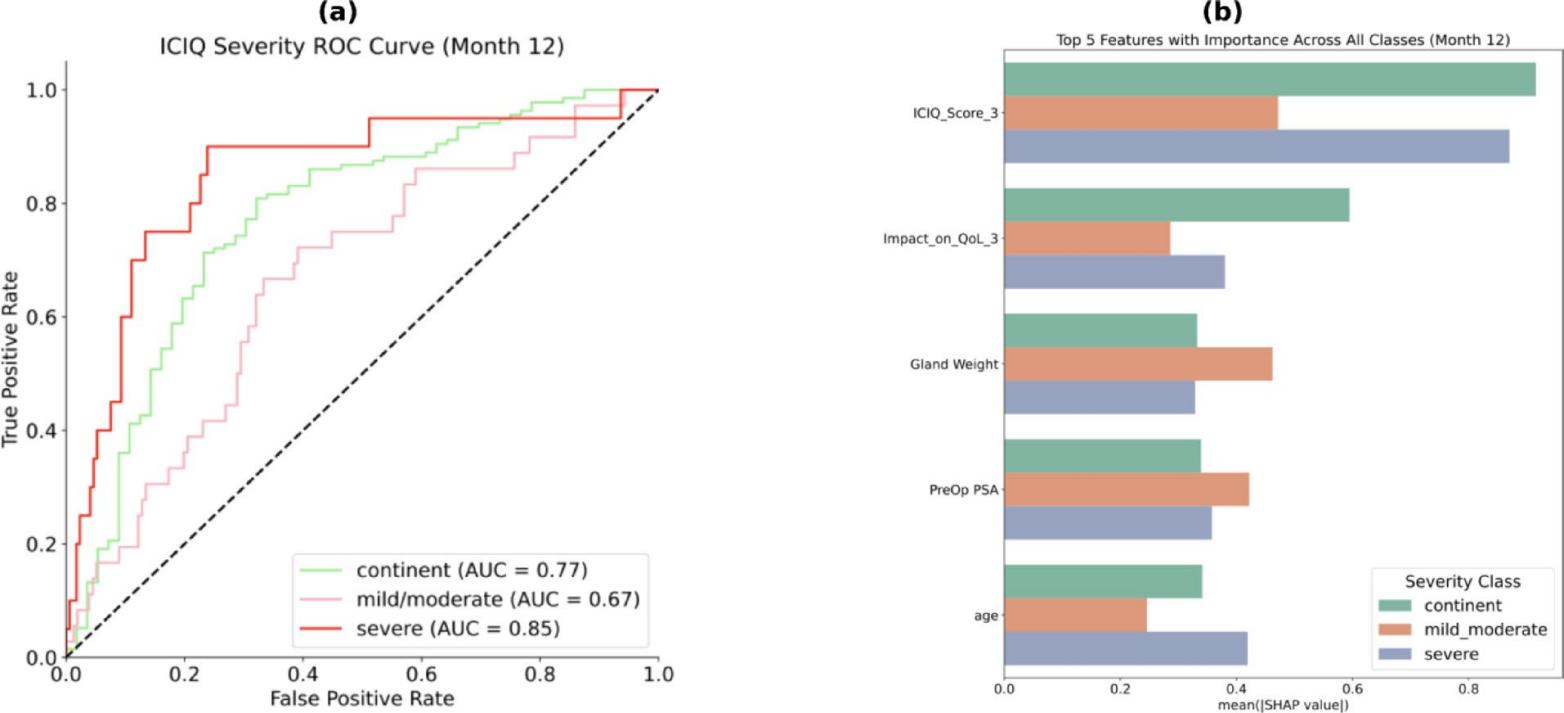
The ROC curves for different ICIQ severity categories at 12 months: continent (AUC = 0.77), mild/moderate (AUC = 0.67), and severe (AUC = 0.85) ICIQ severity classes at 12 months, along with the SHAP values showing feature importance across all severity classes.

The long-term prediction model for ICIQ-UI score severity at 12 months post-surgery demonstrated strong performance across different classification tasks (Figs. 6 and 7).

For the binary classification of Incontinence Status, the model achieved an AUC-ROC of 0.83 (95% CI 0.76–0.88). The Symptoms Severity and Impact on QoL models showed AUC-ROC values of 0.82 (95% CI 0.73–0.89) and 0.86 (95% CI 0.79–0.92), respectively (Fig. 6a). The Symptoms model achieved accuracy of 0.877, precision of 0.345, recall of 0.839, and F1-score of 0.488, while the Impact model showed accuracy of 0.878, precision of 0.323, recall of 0.779, and F1-score of 0.455.

In predicting the ICIQ score severity, the model demonstrated robust performance across severity categories (Fig. 7a). The 3-class severity model achieved per-class AUC-ROC values of 0.77 (continent), 0.67 (mild/moderate), and 0.85 (severe), with accuracy of 0.70, precision of 0.68, and F1-score of 0.68.

The mean absolute SHAP values for the top five features vary across severity categories. The most important features are: Impact on QoL (1.2–1.4), ICIQ Score (0.8–1.2), age (0.4–0.8), Symptoms Severity (0.4–0.6), and BMI (0.2–0.4). Notably, the 3-month Impact on QoL score emerges as the strongest predictor across all severity categories, followed by the 3-month ICIQ score. These results suggest that early post operative outcomes at 3 months are highly predictive of long-term (12-month) urinary continence status. Early Impact on QoL (1.4) appears to be a stronger predictor of long-term QoL than early Symptoms Severity (1.0), especially in moderate cases.

Calibration analysis can be seen in Appendix Figs. 12–17.

**Fig. 8:**
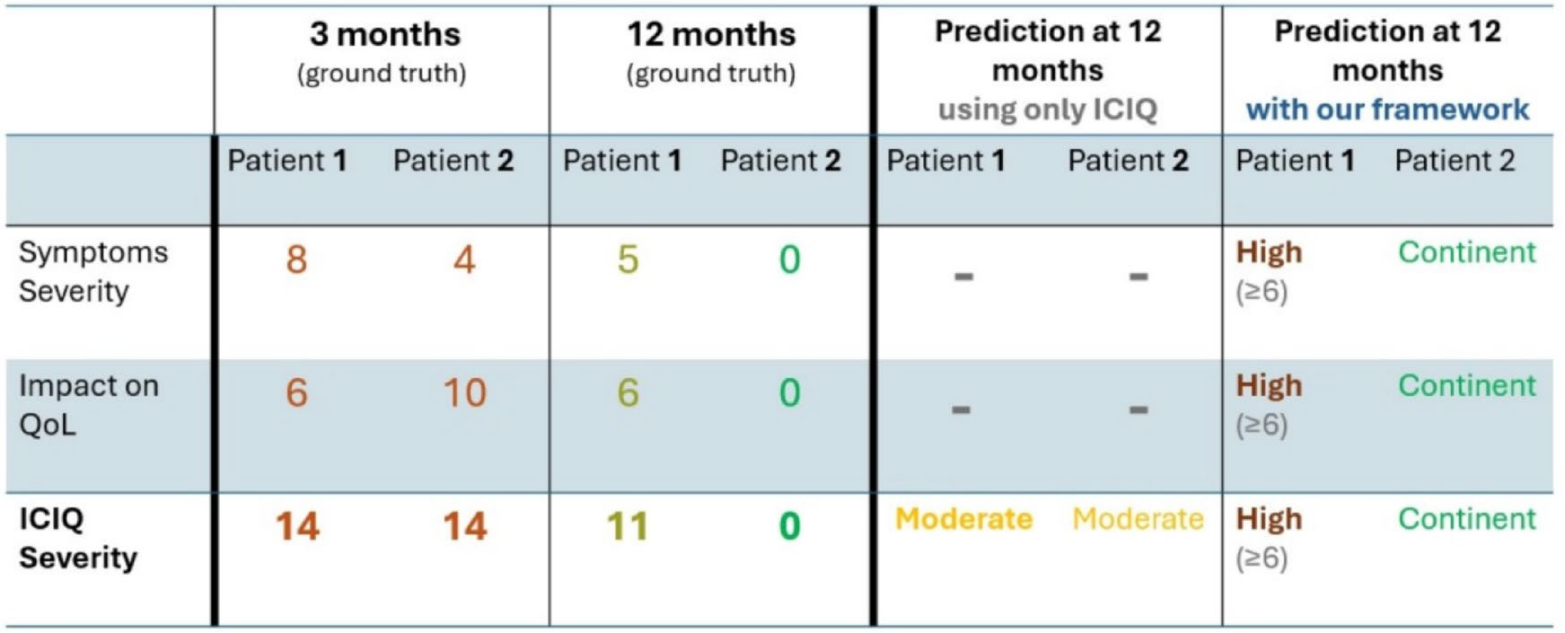
Comparison of prediction approaches using two randomly selected illustrative cases. The figure demonstrates how patients with identical ICIQ severity scores at 3 months can have different symptom patterns and outcomes at 12 months.

### Use case analysis

To demonstrate the clinical utility of our framework, we analyzed four randomly selected illustrative post-RARP cases to highlight the importance of considering multiple dimensions of PPI during recovery prediction.

In the first case analysis (Fig. 8), both patients initially presented with identical ICIQ Severity scores of 14 at three months post-surgery—typically classified as severe incontinence. However, their score composition differed: Patient 1 demonstrated high symptom severity (8) with moderate QoL impact (6), while Patient 2 exhibited lower symptom severity (4) but higher QoL impact (10). These differences proved predictive of their divergent outcomes at 12 months, where Patient 1 remained moderately incontinent (ICIQ score 11), while Patient 2 reached continence (ICIQ score 0). A standard ICIQ-based prediction approach incorrectly classified both patients as “Moderate” at 12 months, failing to differentiate their divergent paths. In contrast, our multidimensional framework correctly predicted high incontinence severity for Patient 1 and full recovery for Patient 2, illustrating the added prognostic value of separating symptom burden and QoL impact.

**Fig. 9:**
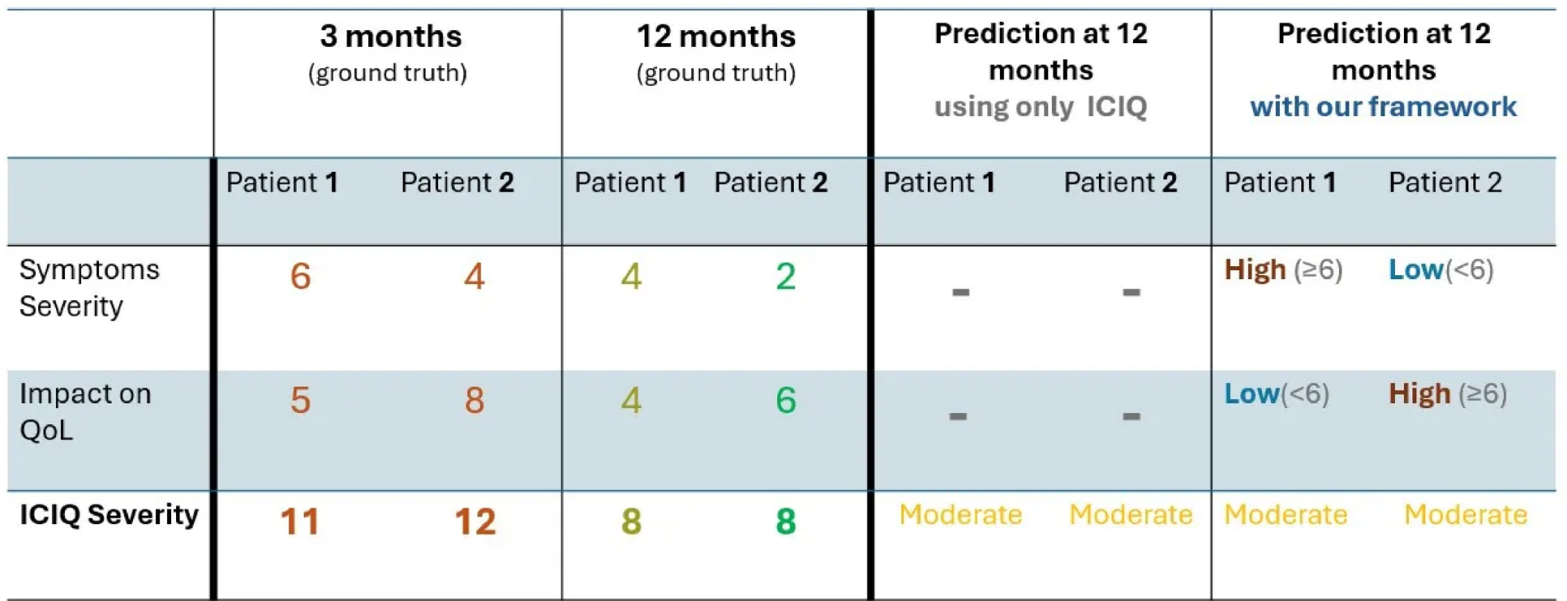
Additional case comparison demonstrating how patients with similar moderate ICIQ severity scores at 3 months can maintain distinct symptom and QoL impact patterns through their recovery journey, despite achieving identical ICIQ scores at 12 months.

A second analysis (Fig. 9) involved two patients with similar moderate ICIQ scores at 3 months (11 and 12) and identical scores at 12 months (8), yet with differing trajectories. Patient 1 presented with higher symptom severity (6) but lower QoL impact (5), while Patient 2 showed lower symptom severity (4) but higher QoL impact (8). At 12 months, these distinct patterns persisted despite convergence in total ICIQ scores: Patient 1’s symptoms improved modestly from 6 to 4, while QoL impact remained relatively stable (5 to 4), whereas Patient 2 experienced a more substantial reduction in symptom severity (4 to 2) but saw QoL impact decrease from 8 to 6. While traditional predictions accurately flagged both as moderate, our model further distinguished between their symptom and QoL patterns, correctly predicting that Patient 1 would maintain high symptom severity with low QoL impact, while Patient 2 would achieve low symptom severity but continue experiencing high QoL impact. This differentiation reflects their unique recovery experiences and suggests that patients may benefit from tailored interventions.

These case comparisons underscore a key insight that total ICIQ score alone does not capture the heterogeneous experiences of PPI recovery. Our framework’s ability to disentangle and predict distinct symptom and impact dimensions enables more accurate and personalized predictions compared to traditional approaches that rely solely on aggregate ICIQ scores. By separately analyzing Impact on QoL and Symptom Severity, our model can identify patients with similar total scores but fundamentally different recovery trajectories, facilitating more targeted interventions and counseling.

### External validation

External validation was performed using data from 728 patients treated in 2022, excluding salvage cases and incorporating MUL measurements. The 2022 cohort showed different outcome distributions compared to the training data (2018–2021). At 3 months, the incontinence rate decreased to 36.3% (265/728) compared to 41.3% in the training cohort. However, among those incontinent at 3 months, 55.6% (138/248) remained incontinent at 12 months, compared to only 28.8% (279/968) in the training cohort, suggesting that while surgical techniques have improved early outcomes, patients experiencing early incontinence may represent a more refractory subgroup.

Model performance across original and fine-tuned validation is summarized in Table 3. For 3-month predictions, the original models showed moderate performance (AUC 0.54–0.55), with fine-tuning on 25% of the 2022 data showing minimal improvement or slight degradation. In contrast, 12-month predictions demonstrated marked improvement with fine-tuning, particularly for binary incontinence prediction (AUC improving from 0.61 to 0.77) and impact on QoL (AUC from 0.58 to 0.85). The differential response to fine-tuning between 3-month and 12-month models suggests that while early outcomes remain challenging to predict even with contemporary data, long-term predictions can be successfully adapted to evolving surgical practices through targeted model updating.

### Supplementary analyses

To assess the risk of overfitting, events-per-variable (EPV) ratios were computed for all models. At 3 months, EPV ranged from 13.4 to 38.5. At 12 months, EPV ranged from 3.6 to 9.9, reflecting the smaller cohort, as seen in Table 4.

**Table 4:** Events per variable (EPV) for each model.

Model	Month	Samples	Features	Classes	Minimum classes	epv
Binary	3	2206	25	2	962	38.5
Severity	3	2206	25	3	336	13.4
Symptoms	3	2206	25	2	584	23.4
Impact	3	2206	25	2	376	15.0
Binary	12	960	28	2	277	9.9
Severity	12	960	28	3	101	3.6
Symptoms	12	960	28	2	155	5.5
Impact	12	960	28	2	143	5.1

**Fig. 10:**
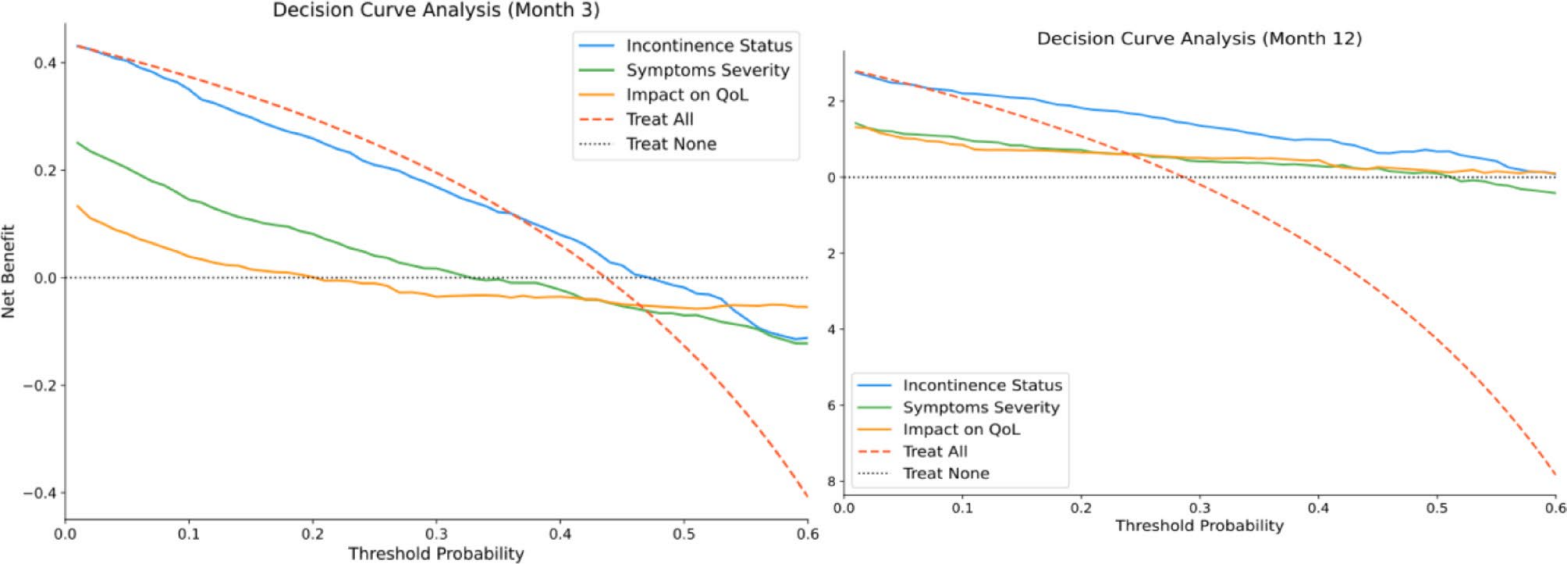
Decision curve analysis.

**Fig. 11:**
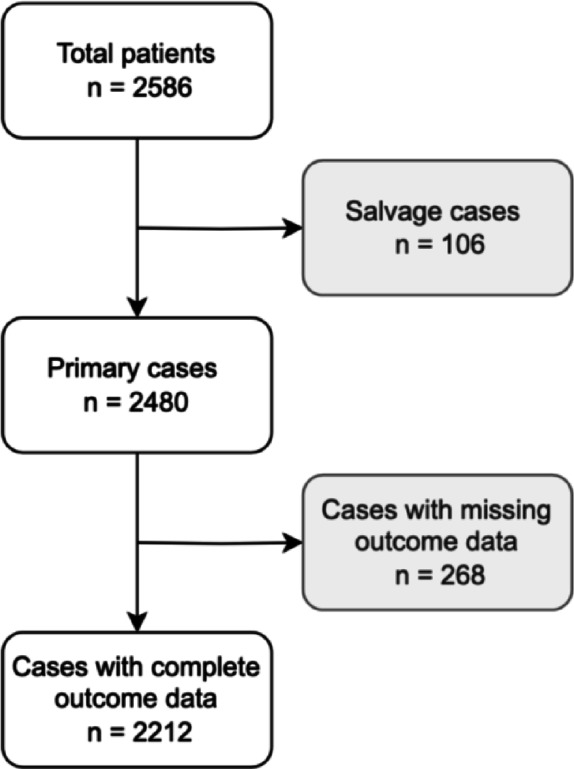
Case disposition chart showing the 2586 total patients across four years (2018–2022), with 106 salvage cases and 268 primary cases without complete outcome data.

Given the high missingness rate for MUL (64%), all models were retrained with MUL excluded entirely. At 3 months, the largest F1 drop was 0.058 (symptoms severity). At 12 months, differences were negligible (< 0.02).Decision curve analysis was performed for the incontinence status models. At 12 months, models demonstrated net benefit above the “treat all” and “treat none” strategies across clinically relevant threshold ranges. At 3 months, net benefit was marginal (Fig. 10).

## Discussion

This study developed machine learning models to predict multiple dimensions of PPI, moving beyond traditional binary classification to capture incontinence status, total ICIQ score, symptom severity, and impact on QoL. This comprehensive approach revealed complex patterns in recovery trajectories and identified novel predictors of long term outcomes. Our work extends prior approaches to PPI prediction, including nomogram-based models^5,6^, binary ML classifiers^10^, and deep learning combined with intraoperative video^7^, by simultaneously predicting multiple outcome dimensions rather than a single endpointOur analysis presents four key findings: First, early prediction of PPI (3 months) remains challenging despite using advanced machine learning techniques. Second, incorporating early postoperative data improves long-term predictions, though not to a high level of precision. Third, subjective patient experiences are as predictive as objective clinical measures. Lastly, identical total ICIQ scores may mask important differences in recovery trajectories, particularly when impact on QoL and symptom severity are considered separately.

The modest performance of our short-term prediction models (AUC-ROC 0.52–0.59) highlights the inherent complexity of early post-surgical recovery. SHAP analysis identified age, BMI, MUL and prostate volume, as the strongest features; however, these did not fully capture the variability in early post-surgical outcomes. This aligns with clinical observations that personalized postoperative care plays crucial roles in early recovery^15^. However, it could also reflect on the absence of granular surgical and early postoperative data in our models. While variables like nerve-sparing status and operative time were included, they may not capture subtle but important technical variations such as urethral sparing, Retzius sparing and bladder neck approaches that influence early continence^16^.

In contrast, the incorporation of 3-month outcome data led to substantially improved 12-month predictions (AUC-ROC 0.82–0.86). This improvement was most notable in predicting impact on QoL (AUC-ROC 0.86 [0.79–0.92]), incontinence status (AUC-ROC 0.83 [0.76–0.88]), and symptom severity (AUC-ROC 0.82 [0.73–0.89]). A novel finding emerged from SHAP analysis: early impact on QoL (SHAP value 1.4) was a stronger predictor of long-term symptom severity than early symptom severity itself (SHAP value 1.0), suggesting that subjective experiences may be more indicative of recovery trajectory than objective symptom measures. However, this finding should be interpreted with caution, as the 3-month ICIQ component scores used as predictors are structurally related to the 12-month outcomes derived from the same questionnaire, which may inflate apparent predictive importance.

Clinical factor analysis revealed a few consistent patterns. At 3 months, patients with shorter MUL, older age, and longer operative times were significantly more likely to experience incontinence, higher symptom severity, and greater impact on QoL (p < 0.01). While BMI and prostate volume showed weak or no significant associations, higher BMI was modestly associated with worse QoL outcomes at both 3 and 12 months. Notably, bilateral nerve-sparing was more common among patients with better continence and symptom profiles at 3 months (p < 0.01), though this trend did not reach significance at 12 months. These results suggest that early incontinence is influenced by anatomical and technical factors, but that long-term outcomes may also depend on recovery dynamics not fully captured in baseline surgical data.

Perhaps our most clinically relevant finding was that patients with identical ICIQ severity scores at 3 months often showed markedly different recovery trajectories at 12 months. Our use case analysis demonstrated that decomposing the ICIQ score into its components, symptom severity and QoL impact, enabled more accurate long-term predictions. In contrast, models using only the total score failed to differentiate between patients with similar scores but distinct trajectories. These findings advocate for a more nuanced approach to early outcome assessment and individualized care planning.

External validation on 2022 data (n = 728) revealed important insights about practice evolution. Three-month incontinence rates improved from 43.5 to 36.3%, suggesting refined surgical techniques. However, a higher proportion (55.6% in 2022 versus 28.8% in 2018–2021) of patients incontinent at 3 months remained incontinent at 12 months. Fine-tuning on 25% of the 2022 cohort yielded differential effects: minimal impact on 3-month predictions but substantial improvements for 12-month outcomes, particularly for QoL impact (AUC improving from 0.58 to 0.85) and binary incontinence (AUC from 0.61 to 0.77). This suggests that while early postoperative outcomes remain inherently unpredictable regardless of model adaptation, long-term recovery patterns can be successfully recalibrated to contemporary surgical practices through targeted model updating.

The study has several important limitations. First, the retrospective single-centre design, while providing a large cohort from an established QA programme, may limit generalisability, including potential selection bias. This was partially mitigated by prospective data collection within our established QA programme. Prospective, multi-centre validation is a priority for future work. Prospective, multi-centre validation is a priority for future work. Second, MUL had the highest missingness rate at 64%, as pre-operative MRI-based MUL measurement was not routinely available throughout the study period; although sensitivity analysis confirmed robustness, this limits MUL-specific conclusions. Third, the absence of the ICIQ-UI’s fourth self-diagnostic item limited our understanding of patients’ perceptions of leakage causes. Fourth, reliance on self-reported outcomes lacks the objectivity of pad weight tests or urodynamic studies^17^, and as d’Altilia et al.^8^ demonstrated, the method of continence assessment—objective pad weight testing versus self-reported pad counts—significantly influences outcome reporting. Fifth, we could not account for variations in post-surgical care and rehabilitation. Sixth, while EPV ratios for binary models were adequate (15.0–38.5 at 3 months, 5.1–9.9 at 12 months), the lower 12-month values may contribute to wider confidence intervals. Finally, our study period may not have captured impacts beyond 12 months. Additionally, our outcome definitions rely on patient-reported pad usage (ICIQ-SF), which, while aligned with recommendations for validated patient-reported outcome measures^8^, is influenced by patient behaviour and may not fully capture objective continence status. Patients continent at 3 months were excluded from 12-month analysis, which may introduce selection bias by not capturing late-onset incontinence.

Several future research directions emerge from this work. First, incorporating post surgical MRI could provide direct information on the structural changes that occurred during surgery. Second, development of a multi-modal prediction framework that integrates real-time intraoperative surgical video analysis with pre-operative data could be combined with our existing prediction framework to create a hybrid model that captures both baseline patient factors and surgical execution quality, potentially revealing which specific surgical maneuvers and techniques are most predictive of positive continence outcomes at both 3 and 12 months post-surgery. Third, extracting simplified decision rules from our trained models could provide clinicians with interpretable thresholds while preserving the insights gained from complex feature interactions.

In conclusion, while early prediction of PPI remains challenging, our study demonstrates that incorporating early postoperative data, particularly patient-reported QoL measures, improves long-term prediction accuracy. Our multidimensional framework enables accurate prediction of both symptom severity and impact on QoL, providing a meaningful advance in PPI outcome assessment and personalized rehabilitation strategies. These findings support the integration of patient-reported outcomes and machine learning approaches in improving post-RARP recovery pathways.

## Figures and Tables

**Figs. 12–17:**
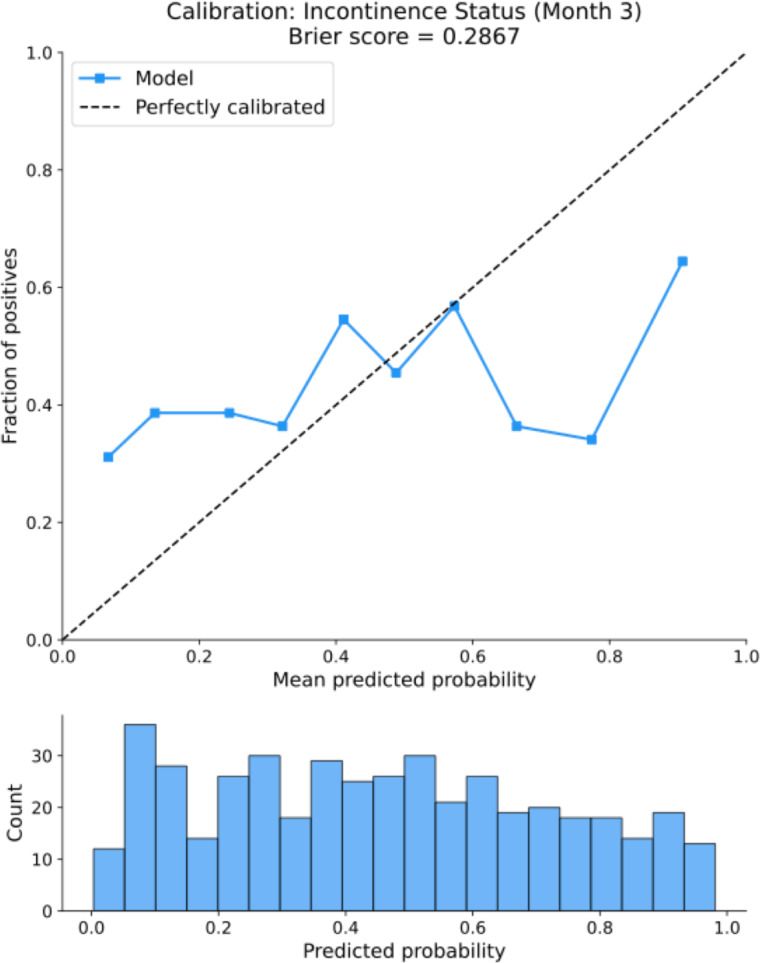

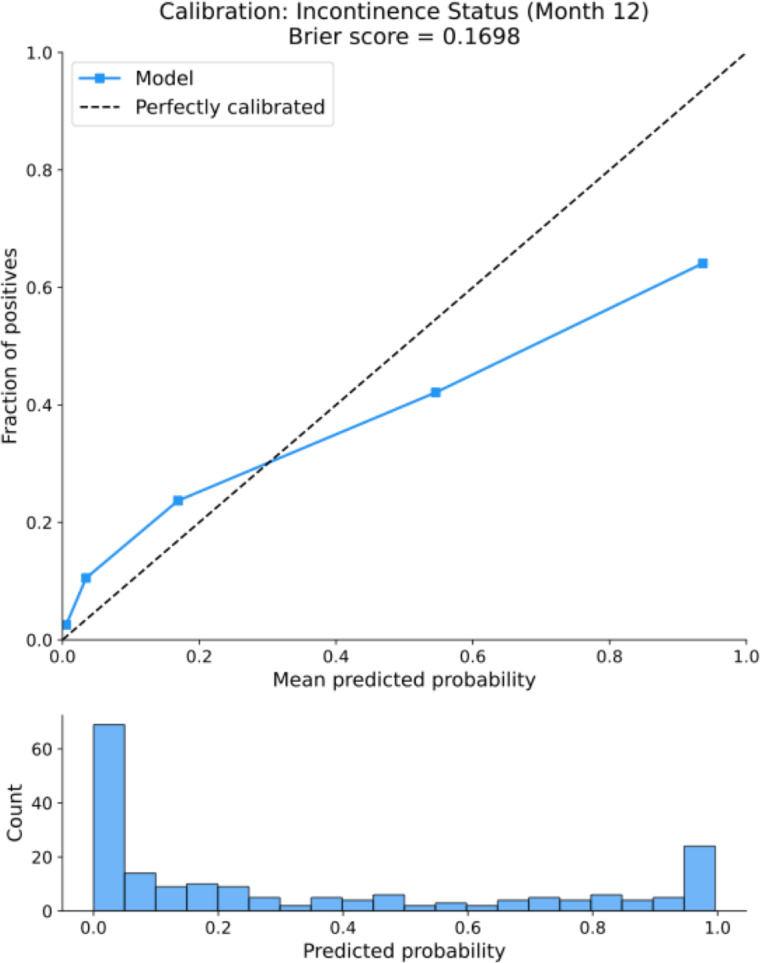

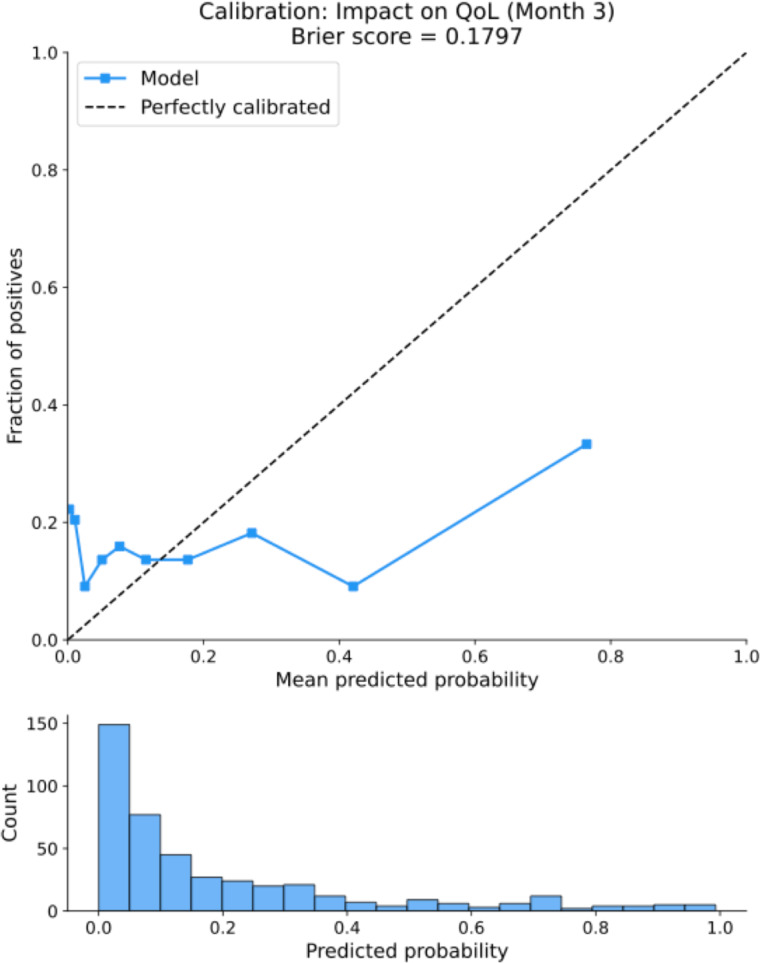

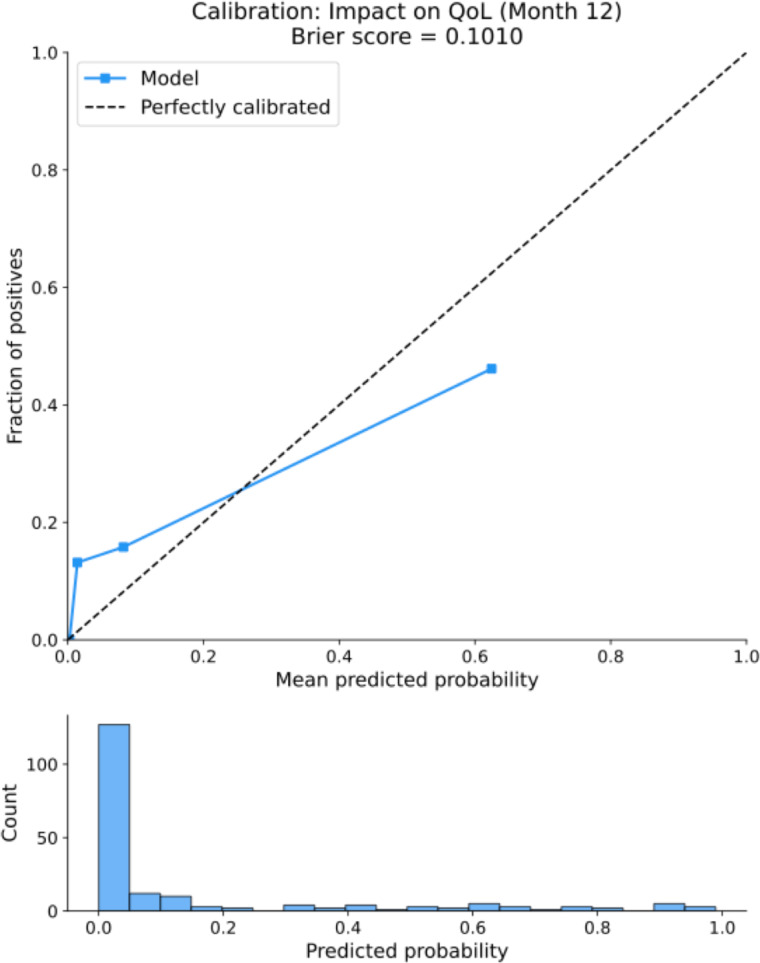

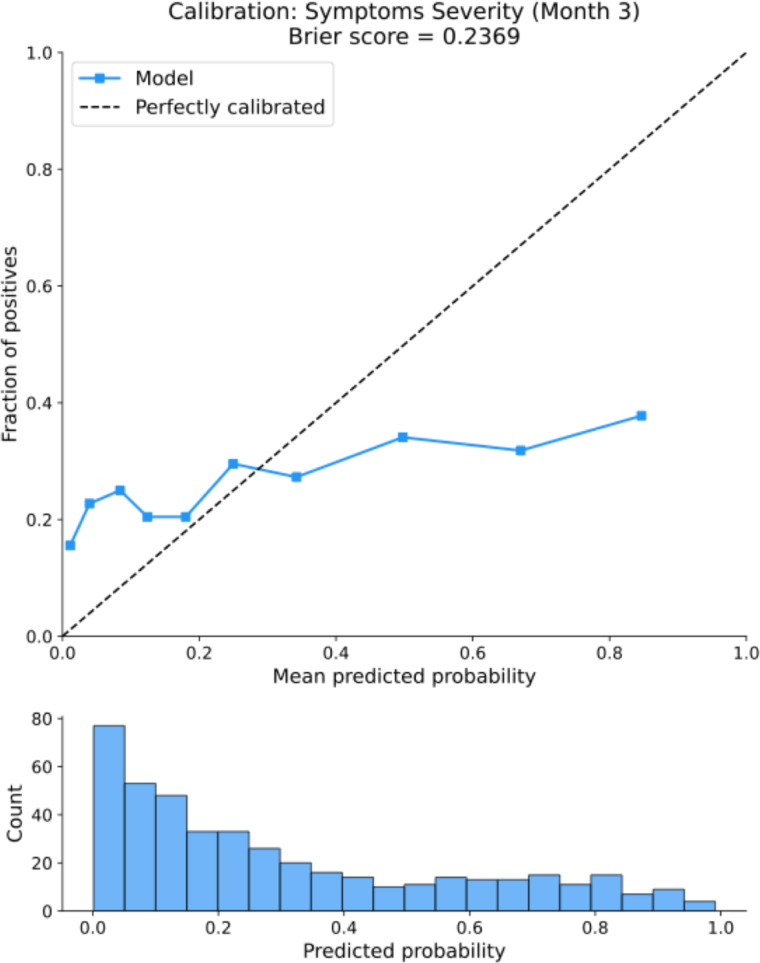

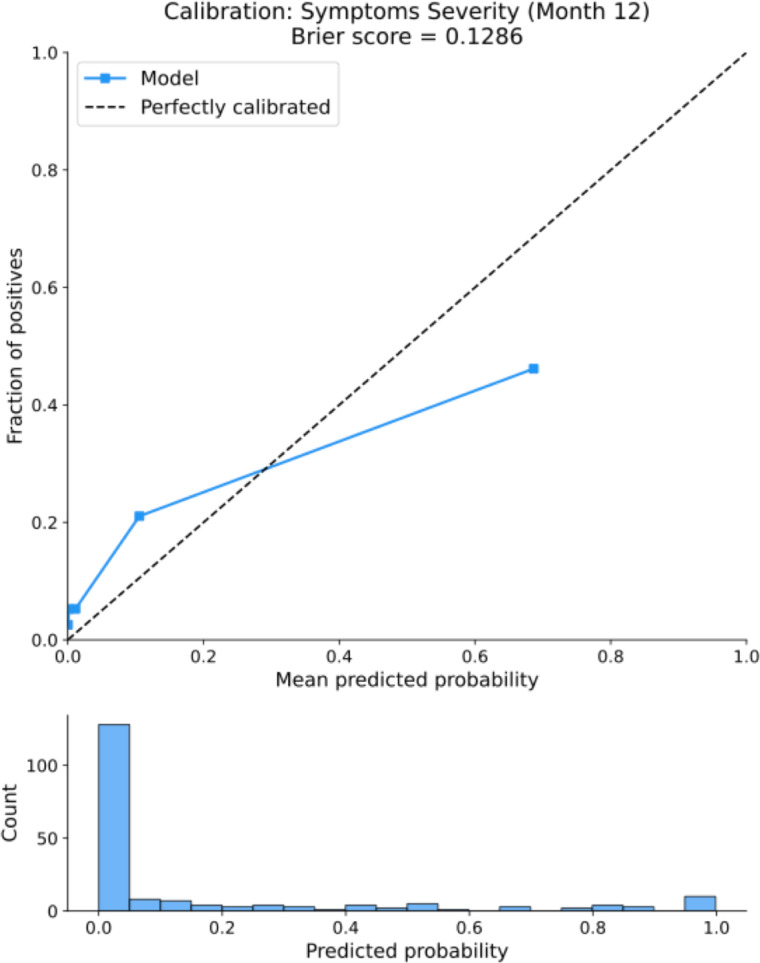
Calibration plots.

## Data Availability

The data that support the findings of this study are not publicly available due to patient privacy restrictions and NHS information governance requirements. De-identified data may be available from the corresponding author upon reasonable request and with permission from University College London Hospitals NHS Foundation Trust Research Ethics Committee.

## Appendix

**Table 5:** Percentage of missing data per feature.

Feature	Percentage missing (%)
Membranous urethral length (MUL)	64.47
Body mass index (BMI)	23.37
Measured blood loss	8.59
Duration of operation	8.45
Gland weight	6.46
Surgeon grade	5.88
Pre-operative PSA	5.79
Continence (12 months)	0.41
Interference (12 months)	0.41
Leak Rate (12 months)	0.41
Continence (3 months)	0.27
Interference (3 months)	0.27
Post-operative ISUP grade	0.09
Pre-operative ISUP grade	0.05
Age	0.00
Surgical margins	0.00
Nerve sparing	0.00
Leak rate (3 months)	0.00

**Table 6:** Patient demographics and clinical characteristics by ICIQ severity classification at 3 months. Continuous features are presented as mean values (± SD), categorical features as count (percentage %), with corresponding p-values for statistical significance.

Feature	ICIQ Severity Classification at 3 months					p-value
	Continent (n = 1244)	Mild (n = 169)	Moderate (n = 457)	Severe (n = 241)	Very Severe (n = 95)	
BMI (kg/m^2^)	28.00 ± 3.90	28.00 ± 4.20	28.00 ± 3.90	28.00 ± 4.40	28.00 ± 4.60	0.70
MUL (mm)	15.00 ± 3.20	14.00 ± 3.00	14.00 ± 3.00	13.00 ± 3.40	14.00 ± 2.80	< 0.01
PreOp PSA (ng/mL)	8.40 ± 6.40	9.70 ± 7.40	9.40 ± 7.00	9.40 ± 5.70	6.70 ± 3.00	0.24
MRI prostate volume (cc)	40.00 ± 19.00	47.00 ± 27.00	43.00 ± 23.00	53.00 ± 40.00	44.00 ± 17.00	0.24
Age (years)	62.00 ± 7.10	64.00 ± 6.20	63.00 ± 7.00	64.00 ± 8.80	64.00 ± 7.90	< 0.01
Measured blood loss (L)	0.15 ± 0.37	0.13 ± 0.34	0.17 ± 0.40	0.18 ± 0.39	0.14 ± 0.39	0.67
Duration of operation (h)	2.80 ± 1.40	3.30 ± 1.50	2.80 ± 1.30	3.10 ± 1.40	3.20 ± 1.50	< 0.01
ISUP PreOp grade						
Grade 1	142 (11.4%)	11 (6.5%)	49 (10.7%)	27 (11.2%)	10 (10.5%)	0.44
Grade 2	733 (58.9%)	106 (62.7%)	261 (57.1%)	133 (55.2%)	53 (55.8%)	0.55
Grade 3	251 (20.2%)	31 (18.3%)	97 (21.2%)	54 (22.4%)	19 (20.0%)	0.87
Grade 4	78 (6.3%)	11 (6.5%)	33 (7.2%)	12 (5.0%)	10 (10.5%)	0.42
Grade 5	39 (3.1%)	10 (5.9%)	17 (3.7%)	15 (6.2%)	3 (3.2%)	0.11
ISUP PostOp grade						
Grade 1	105 (8.4%)	4 (2.4%)	32 (7.0%)	18 (7.5%)	2 (2.1%)	0.02
Grade 2	791 (63.6%)	118 (69.8%)	294 (64.3%)	139 (57.7%)	56 (58.9%)	0.12
Grade 3	270 (21.7%)	36 (21.3%)	108 (23.6%)	67 (27.8%)	27 (28.4%)	0.18
Grade 4	19 (1.5%)	3 (1.8%)	6 (1.3%)	4 (1.7%)	3 (3.2%)	0.77
Grade 5	57 (4.6%)	8 (4.7%)	17 (3.7%)	13 (5.4%)	7 (7.4%)	0.59
Nerve sparing						
Bilateral	558 (44.9%)	66 (39.1%)	183 (40.0%)	76 (31.5%)	34 (35.8%)	< 0.01
Unilateral	443 (35.6%)	63 (37.3%)	152 (33.3%)	73 (30.3%)	28 (29.5%)	0.34
Unknown	9 (0.7%)	3 (1.8%)	5 (1.1%)	4 (1.7%)	0 (0.0%)	0.38
Surgeon grade						
Consultant	902 (72.5%)	130 (76.9%)	347 (75.9%)	183 (75.9%)	70 (73.7%)	0.47
SpR	261 (21.0%)	37 (21.9%)	81 (17.7%)	42 (17.4%)	23 (24.2%)	0.33

**Table 7:** Patient demographics and clinical characteristics by ICIQ severity classification at 12 months. Continuous features are presented as mean values (± SD), categorical features as number (percentage), with corresponding p-values for statistical significance.

Feature	ICIQ severity classification at 12 months					p-value
	Continent (n = 677)	Mild (n = 56)	Moderate (n = 121)	Severe (n = 73)	Very severe (n = 27)	
BMI (kg/m^2^) (mean ± SD)	28.00 ± 4.00	28.00 ± 4.20	27.00 ± 4.10	29.00 ± 5.00	28.00 ± 4.20	0.57
MUL (mm) (mean ± SD)	14.00 ± 2.90	14.00 ± 3.40	13.00 ± 3.40	14.00 ± 4.10	16.00 ± 3.30	0.70
PreOp PSA (ng/mL)	9.60 ± 7.20	8.30 ± 7.00	9.20 ± 5.00	8.40 ± 5.00	7.40 ± nan	0.66
MRI prostate volume (cc)	46.00 ± 25.00	47.00 ± 33.00	47.00 ± 44.00	51.00 ± 24.00	32.00 ± 0.71	0.49
Age (years)	63.00 ± 7.10	64.00 ± 5.40	64.00 ± 7.20	63.00 ± 7.60	62.00 ± 15.00	0.61
Measured blood loss (L)	0.15 ± 0.38	0.13 ± 0.34	0.14 ± 0.35	0.33 ± 0.48	0.12 ± 0.33	0.02
Duration of operation (h)	2.90 ± 1.30	3.60 ± 1.70	2.80 ± 1.30	3.30 ± 1.30	3.70 ± 1.60	< 0.01
ISUP PreOp grade (n (%))						
Grade 1	64 (9.5%)	4 (7.1%)	11 (9.1%)	15 (20.5%)	2 (7.4%)	0.04
Grade 2	404 (59.7%)	38 (67.9%)	54 (44.6%)	34 (46.6%)	19 (70.4%)	< 0.01
Grade 3	133 (19.6%)	11 (19.6%)	34 (28.1%)	17 (23.3%)	5 (18.5%)	0.31
Grade 4	48 (7.1%)	1 (1.8%)	11 (9.1%)	4 (5.5%)	1 (3.7%)	0.42
Grade 5	28 (4.1%)	2 (3.6%)	11 (9.1%)	3 (4.1%)	0 (0.0%)	0.12
ISUP PostOp grade (n (%))						
Grade 1	41 (6.1%)	1 (1.8%)	8 (6.6%)	5 (6.8%)	1 (3.7%)	0.70
Grade 2	436 (64.4%)	35 (62.5%)	68 (56.2%)	45 (61.6%)	16 (59.3%)	0.53
Grade 3	160 (23.6%)	17 (30.4%)	32 (26.4%)	19 (26.0%)	10 (37.0%)	0.43
Grade 4	10 (1.5%)	0 (0.0%)	5 (4.1%)	1 (1.4%)	0 (0.0%)	0.20
Grade 5	30 (4.4%)	3 (5.4%)	8 (6.6%)	3 (4.1%)	0 (0.0%)	0.63
Nerve sparing (n (%))						
Bilateral	257 (38.0%)	21 (37.5%)	42 (34.7%)	26 (35.6%)	10 (37.0%)	0.97
Unilateral	225 (33.2%)	22 (39.3%)	39 (32.2%)	20 (27.4%)	6 (22.2%)	0.48
Unknown	12 (1.8%)	0 (0.0%)	0 (0.0%)	0 (0.0%)	0 (0.0%)	0.29
Involvement of training (n (%))						
No	521 (77.0%)	41 (73.2%)	89 (73.6%)	55 (75.3%)	20 (74.1%)	0.90
Yes	119 (17.6%)	15 (26.8%)	25 (20.7%)	14 (19.2%)	6 (22.2%)	0.48
3-month outcomes (median ± IQR)						
Symptoms severity	6.00 ± 1.90	7.00 ± 2.30	6.00 ± 2.00	8.00 ± 1.90	10.00 ± 1.90	< 0.01
Impact on QoL	4.00 ± 2.40	5.50 ± 2.90	7.00 ± 2.50	9.00 ± 2.40	10.00 ± 2.00	< 0.01
ICIQ Score	1.00 ± 0.79	2.00 ± 0.96	2.00 ± 1.30	3.00 ± 1.50	4.00 ± 1.40	< 0.01

## References

[1] Bernardes, M. F. V. G. et al. Impact of urinary incontinence on the quality of life of individuals undergoing radical prostatectomy. *Rev. Lat. Am. Enfermagem.* **27**, e3131 (2019). 30916232 10.1590/1518-8345.2757.3131 PMC6432995

[2] Borregales, L. D. et al. Trifecta’after radical prostatectomy: Is there a standard definition?. *BJU Int.* **112**(1), 60–67 (2013). 23759009 10.1111/bju.12002

[3] Sacco, E. et al. Patient pad count is a poor measure of urinary incontinence compared with 48-h pad test: Results of a large-scale multicentre study. *BJU Int.* **123**(5A), E69–E78 (2019). 30253042 10.1111/bju.14566

[4] Nguyen, H., Butow, P., Dhillon, H. & Sundaresan, P. A review of the barriers to using patient-reported outcomes (PROs) and patient-reported outcome measures (PROMs) in routine cancer care. *J. Med. Radiat. Sci.* **68**(2), 186–195 (2021). 32815314 10.1002/jmrs.421 PMC8168064

[5] Pinkhasov, Ruben M. et al. Prediction of incontinence after robot-assisted radical prostatectomy: development and validation of a 24-month incontinence nomo gram. *Cancers* **14**(7), 1644 (2022). 35406416 10.3390/cancers14071644 PMC8997126

[6] Shen, C. et al. Nomogram predicting early urinary incontinence after radical prostatectomy. *BMC Cancer* **24**(1), 1095 (2024). 39227825 10.1186/s12885-024-12850-1 PMC11373233

[7] Nakamura, W. et al. Combination of deep learning and ensemble machine learning using intraoperative video images strongly predicts recovery of urinary continence after robot-assisted radical prostatectomy. *Cancer Reports* **6**(9), e1861. 10.1002/cnr2.1861 (2023). 37449339 10.1002/cnr2.1861 PMC10480482

[8] d’Altilia, N. et al. A matched-pair analysis after robotic and retropubic radical prostatectomy: a new definition of continence and the impact of different surgical techniques. *Cancers* **14**, 4350. 10.3390/cancers14184350 (2022). 36139511 10.3390/cancers14184350 PMC9496957

[9] Li, J. et al. An artificial intelligence method for predicting postoperative urinary incontinence based on multiple anatomic parameters of MRI. *Heliyon* **9**(10), e20337. 10.1016/j.heliyon.2023.e20337 (2023). 37767466 10.1016/j.heliyon.2023.e20337 PMC10520312

[10] Amparore, D. et al. Development of a machine learning algorithm to predict the risk of incontinence after robot-assisted radical prostatectomy. *J. Endourol.* **38**(8), 871–878. 10.1089/end.2024.0057 (2024). 38512711 10.1089/end.2024.0057

[11] Cathcart, P. et al. Achieving quality assurance of prostate cancer surgery during reorganisation of cancer services. *Eur. Urol.* **68**(1), 22–29 (2015). 25770482 10.1016/j.eururo.2015.02.028

[12] Avery, K. et al. Iciq: A brief and robust measure for evaluating the symptoms and impact of urinary incontinence. *Neurourol. Urodyn.* **23**(4), 322–330 (2004). 15227649 10.1002/nau.20041

[13] Iciq — the international consultation on incontinence questionnaire. https: //iciq.net/. (Accessed 30 August 2024).

[14] Klovning, A., Avery, K., Sandvik, H. & Hunskaar, S. Comparison of two questionnaires for assessing the severity of urinary incontinence: The ICIQ UI SF versus the Incontinence Severity Index. *Neurourol. Urodyn.* **28**(5), 411–415 (2009). 19214996 10.1002/nau.20674

[15] Du, C., Li, H., Qu, L., Li, Y. & Bao, X. Personalized nursing care improves psychological health, quality of life, and postoperative recovery of patients in the general surgery department. *Int. J. Clin. Exp. Med.* **12**(7), 9090–9096 (2019).

[16] Katsimperis, Stamatios et al. Surgical techniques to preserve continence after robot-assisted radical prostatectomy. *Front. Surg.* **10**, 1289765 (2023). 38026481 10.3389/fsurg.2023.1289765 PMC10655003

[17] Uroweb - European Association of Urology. (n.d.). EAU Guidelines on the Management of Non-neurogenic Male LUTS - INTRODUCTION - Uroweb. https://uroweb.org/guidelines/management-of-non-neurogenic-male-luts.

